# Measurement Equivalence and Feasibility of the Electronic and Paper Versions of the POSAS, EQ-5D, and DLQI: A Randomized Crossover Trial

**DOI:** 10.3390/ebj5040030

**Published:** 2024-10-11

**Authors:** Jill Meirte, Nick Hellemans, Ulrike Van Daele, Koen Maertens, Lenie Denteneer, Mieke Anthonissen, Peter Moortgat

**Affiliations:** 1 Department of Rehabilitation Sciences and Physiotherapy REVAKI-MOVANT, Faculty of Medicine and Health Sciences, University of Antwerp, 2610 Wilrijk, Belgium; 2 Oscare, Organization for Burns, Scar After-Care and Research, 2170 Antwerp, Belgium; 3 Awell Health, 1000 Brussel, Belgium; 4 Department of Clinical and Lifespan Psychology, Vrije Universiteit Brussel, 1000 Brussels, Belgium; 5 Horacio Oduber Hospital, Oduber Boulevard 1, Oranjestad 0000 AW, Aruba

**Keywords:** patient-reported outcome measures (PROMs), mode of administration, scars, scar quality, quality of life, paper/electronic PROMs, agreement

## Abstract

Patient-reported outcome measures (PROMs) are crucial within person-centered care. The use of electronic PROMs (ePROMs) is increasing and multiple advantages have been described. The Patient and Observer Scar Assessment Scale (POSAS) is a validated paper questionnaire to assess patient-reported scar quality in the burn and scar population. In burn and scar rehabilitation, quality of life questionnaires such as the Euroqol 5 Dimensions 5 level (EQ-5D-5L) and the Dermatology Life Quality Index (DLQI) allow us to measure physical and psychosocial impact. The goal of this research was to compare the equivalence of the electronic versions of the POSAS, the EQ-5D-5L, and the DLQI with their original paper counterparts. To ensure the psychometric properties of the electronic versions, we assessed the equivalence of scores, the differences in completion time, and patients’ preferred mode and ease of use. We used a randomized crossover design using a within-subject comparison of the formats of the questionnaires. Participants aged over 18 with a scar were recruited from an outpatient after-care and research center for burns and scars in Antwerp, Belgium. The equivalence of the electronic questionnaires POSAS, EQ-5D-5L, and DLQI is assumed based on the findings of this study. Completion times were faster for all the electronic versions but only statistically different (*p* = 0.002) for the electronic version of the EQ-5D-5L. The number of missing answers could be reduced to 0. The electronic assessment was preferred in >75% of the cases and subjects found it easy to use, and a tool that could improve the quality of care. Our findings support the electronic delivery of POSAS, EQ-5D, and DLQI, within the burn and scar population.

## 1. Introduction

At the heart of our healthcare delivery system lies the patient, whose voice and experiences are pivotal in patient-centered or person-centered care (PCC) [1,2]. Patient-reported outcome measures (PROMs) serve as crucial tools for soliciting information from patients and detecting vital details about their needs and therapeutic progress. Their significance in both the care process and interventions for scar management cannot be overstated. Recent qualitative research underscores the necessity of integrating person-centered scar assessment into routine follow-ups, emphasizing the importance of establishing a clear purpose for scar assessment and providing transparent feedback loops to patients. Patients particularly appreciate the opportunity to address psychosocial impact and rehabilitation through the use of such tools [3].

A previous call to action advocated for the routine assessment of the quality of life during patient follow-ups [4], and this was further bolstered by a recommendation to conduct holistic evaluations of scar management interventions. This ensures that future evidence-based decisions are made in a patient-centered manner [5]. The Patient and Observer Scar Assessment Scale (POSAS) with its Patient Scar Assessment Scale for patient opinion is a world-wide known and used PROM for the follow-up of patients with (burn) scars [6]. The POSAS assesses scar quality with physical scar features and scar appearance and is considered to be a valid patient-oriented questionnaire for guiding scar treatment options in patients with scars [7]. The Dermatology Life Quality Index (DLQI) [8] is the most commonly used dermatology-specific quality of life (QOL) measure in clinical trials [9]. Within the scar population, it is also one of the most commonly used QOL questionnaires [10]. The Euroqol 5 Dimensions (EQ-5D) is a widely used generic QOL instrument [11]. Within the burn population, this questionnaire is one of the suggested established QOL measures for defining the recovery of QOL [12]. It includes relevant health domains, is applicable in all kinds of injury populations and in different age ranges, provides a link to utility scores, and has several practical advantages (e.g., brevity and availability in different languages) [10]. The brevity and simplicity of both POSAS, EQ-5D, and DLQI is an explanation for the popularity of the measures both clinically and within research.

PROMs designed for patients with burns and scars were originally created and administered via paper questionnaires. The modes of administration (MOAs) for collecting patient-reported outcomes commonly include paper or electronic formats (e.g., tablets). However, in recent years, there has been a rapid transition from paper-based to electronic patient-reported outcome measures (ePROMs). While paper-pen questionnaires were previously favored for their ease of use and widespread acceptance among patients, they also presented numerous limitations [13]. Conversely, ePROMs offer several advantages over traditional paper-based MOAs. They are generally preferred more, entail lower costs, enhance data quality, maintain comparable or quicker completion times, require less administration time, and can facilitate clinical decision making when coupled with appropriate symptom management [14].

To ensure high-quality measurements of PROMs, the implementation of ePROMs should be well planned and executed [15,16]. Most PROMs were developed and validated on paper, thus migrating them to an electronic version could alter the clinical properties and psychometric properties cannot be assumed across administration modes. When altering the mode of administration from paper to tablet, moderate modifications (such as splitting an item across multiple screens or requiring the use of a scroll bar to see all the items) generally require equivalence testing together with usability testing [17] since ePROMs may not always yield the same results electronically. Guidelines are available for developing and validating ePROMs [18,19]. When considering the validation of a new ePROM, the extent to which psychometric testing needs to be reevaluated depends on the types of modifications made. When migration without substantive or major changes is performed, electronic versions generally provide comparable data [20]. The decision regarding the need for the generation of additional evidence depends on the amount of available supporting evidence demonstrating whether the change has affected the PROM’s measurement properties [17]. It is recommended to empirically evaluate score equivalence and accordance with modes. In recent research, there are only a handful of papers on ePROM evaluation in patients with scars in the after-care setting. In an outpatient cosmetic surgery clinic, both patients and therapists preferred electronic MOA over paper, and ePROMs could possibly replace some follow-up appointments leading to timesaving of all the parties involved [21]. In a recent study performed in the Netherlands, 84% of patients with burn injuries had no problems with online questionnaires as long as it took less than 15 min to administer [22]. For the electronic versions of the POSAS, DLQI, and EQ-5D-5L, we lack info about the equivalence, feasibility, and usability in the burn and scar population.

At Oscare, an outpatient after-care and research center for patients with scars and burns, located in Antwerp, we acknowledged that our paper-pen instruments were time-consuming and their use in a clinical context was limited. We, therefore, conducted a randomized crossover trial to test the score equivalence of the paper and electronic versions of the POSAS, EQ-5D-5L, and DLQI for individual items, subscale scores, and the total score. We hypothesized equivalence between the two administration modes. Differences in completion time, data quality, and administration times between both MOAs were also a measure of interest. Patients’ thoughts about ePROs such as the ease of use, preferred MOA, and perceived benefit were assessed.

## 2. Materials and Methods

### 2.1. Study Design

This study employed a single-center randomized, two-arm crossover design. This equivalence and feasibility study used a within-subject comparison of the 2 formats of the questionnaires. The study is reported in accordance with CONSORT crossover guidelines [23]. Crossover design is the preferred method for equivalence testing [20]. Patients were randomized into either of two groups: (1) a group that filled in the paper questionnaires first (paper-first, PF) and (2) a group that filled in the electronic questionnaires first (electronic-first, EF). Both groups completed both MOAs.

**Figure 1:**
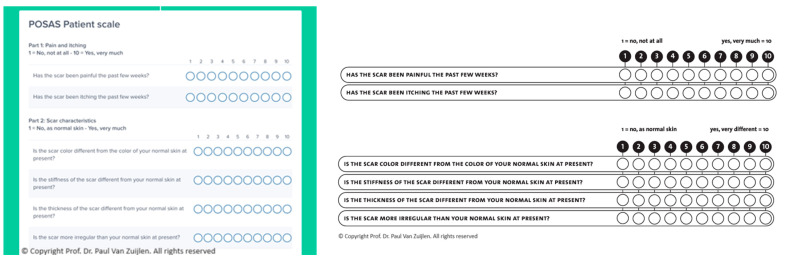
(**Left**) visualizes part of the electronic versions of the ePOSAS in the digital pathway, and (**right**) shows a snapshot of the paper version of the POSAS [24].

**Figure 2:**
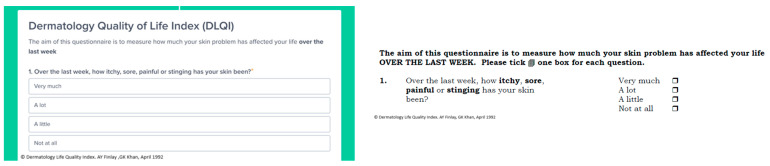
(**Left**) visualizes part of the electronic version of the eDLQI in the digital pathway, and (**right**) shows a snapshot of the paper version of the DLQI [8] illustrating that only minor modifications were made in the electronic migration process [16].

To develop the electronic versions of the POSAS, EQ-5D-5L, and DLQI, we used a cloud service from Awell (https://awellhealth.com, accessed on 29 June 2024). They developed a Software as a Service (SAAS) where care pathways are digitized and patient data are collected electronically via their channel of choice (e.g., tablet or computer). In Figure 1 and Figure 2 snapshots of the electronic and paper versions of the questionnaires are illustrated.

We used the Dutch versions of the questionnaires; for reasons of clarity, the English versions are shown here.

### 2.2. Study Participants and Setting

The research was conducted from November 2020 to May 2023, with interruptions due to COVID-19. It took place in Oscare, a scar after-care and research center in Antwerp, Belgium, representing both rural and urban populations. Patients were asked to participate during their visit to the after-care outpatient clinic. Eligible patients were all adults aged 18 or over with scars, who had the ability to read written and understand speaking Dutch. Scars of all ages, types, and causes could be included in this study. Exclusion criteria were patients with a coexisting medical or secondary dermatological condition of considerable severity as determined by the investigator and those who were not able to read and/or understand Dutch. All the participants who agreed to participate gave written informed consent. The study was approved by the local Ethics Committee of ZNA Antwerp ID N° 5420.

### 2.3. Intervention and Procedure

Patients were screened for inclusion in the study. Eligible patients, who agreed to share contact details, were contacted by the research team and informed about the study and were given more information in person. Randomization was performed automatically when the patient’s information and demographic information were entered into the research pathway. A piece of JavaScript (JS) code randomly allocated the subject into one of two groups. There was no involvement, besides confirming the randomization, of the researchers in the randomization procedure. Our randomization procedure did not take equal randomization groups into account. Neither the research staff nor the participants were blinded to the result of randomization.

Eligible patients were enrolled in our electronic research pathway, which comprised five steps: (1) start-up involving randomization and obtaining informed consent (IC); (2) intake for collecting sociodemographic data, scar data, and assessing technology acceptance and prior knowledge; (3–4) the completion of three questionnaires in both MOAs; and (5) a satisfaction survey evaluating the preferred MOA and attitudes towards ePROMs utilizing a 5-point Likert scale. To minimize any potential learning or memorization effects that could influence outcome measures, such as completion time or score equivalence in the second MOA, a washout period of at least 15 min was implemented between administrations to mitigate recall bias and carry-over effects. The entire research procedure for data capturing was conducted electronically: the patients filled out forms and questionnaires on a tablet (Samsung SM-T580) and on pen-and-paper, while researchers performed certain actions, recorded the data, or completed forms on their personal computer or tablet. Both modes were completed within a single outpatient visit.

### 2.4. Outcome Measures: Patient-Reported Outcome Measures

The POSAS version 2.0, a scar-specific questionnaire designed to evaluate the construct scar quality, was utilized [24]. This scale is recognized as a valid and reliable tool for scar assessment and is a 13-item instrument with a 7-item Observer Scar Assessment Scale (OSAS) and 7-item Patient Scar Assessment Scale (PSAS). This PSAS comprises six items, each rated on a 1 to 10-point scale: pain, itch, color, stiffness, thickness, and irregularity, resulting in a total sum score ranging from 6 to 60. A higher score indicates poorer scar quality [7,24,25]. Additionally, the PSAS includes an overall opinion score rated on a 10-point scale with scores ranging between 1 (most beautiful scar) and 10 (worst possible scar). The PSAS provides two scores: the total sum of scores and the overall opinion of the scar. The paper PSAS shows good test–retest reliability [26]. While a newer version, POSAS version 3.0, is now available, it was not accessible at the initiation of this study.

The DLQI is a validated dermatology-specific questionnaire designed for routine use assessing the construct health-related quality of life in patients with various dermatologic conditions [8,9]. It contains 10 items related to itch and pain, self-consciousness, hindrance in shopping and household, choice of clothing, social activities, sports activities, work, relationships with partners and friends, sexual activity, and treatment impact. For the total score, each question is scored on a four-point Likert scale (very much = 3 | a lot = 2 | a little = 1, not at all = 0), and DLQI is calculated by adding the score of each question (0–3) resulting in a maximum of 30 and a minimum of 0. The higher the score, the more quality of life is impaired. The 10-item DLQI can also be analyzed in 6 subscales by adding up the scores of one or two items depending on the subscale: Symptoms and feelings (items 1 and 2), Daily activities (items 3 and 4), Leisure (items 5 and 6), Work/School (item 7), Personal relationships (item 8 and 9), and Treatment (item 10) [27].

The EQ-5D-5L is a generic measure of health-related quality of life that consists of a descriptive health state and an EQ visual analog scale (VAS) EQVAS score. The descriptive system has five dimensions/subscales of health: (1) Mobility, (2) Self-care, (3) Usual activities, (4) Pain and discomfort, and (5) Anxiety and depression, each with five levels of problems [28,29]. Each dimension has 5 levels: no problems = 1 | slight problems = 2 | moderate problems = 3 | severe problems = 4 | and extreme problems = 5. The digits for the five dimensions can be combined into a 5-digit number that describes the patient’s health state with 11111 indicating no problems on any of the five dimensions. The EQ VAS records the patient’s self-rated health on a vertical visual analog scale where the endpoints are labeled ‘the best health you can imagine’ = 100 and ‘the worst health you can imagine = 0’. The EQ-5D-5L health state can be converted into an index value that can facilitate the calculation of quality-adjusted life years (QALYs), designed for economic evaluation, and is presented in country-specific value sets. The calculation of this index value was not integrated into the electronic calculations and was performed via the “Crosswalk Index Value Calculator” provided by EuroQol for both MOAs [30,31]. A value set consists of weights that can convert each EQ-5D health profile into a value on a scale anchored at 1 (meaning full health) and 0 (meaning a state as bad as being dead) [32].

For all the questionnaires, written or oral approval of use was obtained: DLQI license ID CUQol1933, EQ-5D license ID 21818, and for POSAS, oral approval via personal communication of the creator PVZ was obtained.

Besides the equivalence of the scores between MOAs, the differences in completion times, data quality, the preferred MOA (paper, electronic, or undecided) and the subject’s thoughts towards ePROMs were also assessed. The latter was performed via a 5-point Likert scale (1 = completely disagree, 5 = completely agree).

Completion time was defined as the amount of time necessary for a respondent to complete a questionnaire and was chronometrically measured. Data quality or data (in)completion was defined as the number of incomplete responses and ambiguous data entries (e.g., two answer options ticked).

### 2.5. Statistical Analysis

#### 2.5.1. Sample Size and Power

Sample size calculation for this study was carried out based on the crossover study design. With a power of 80%, a target intraclass correlation coefficient (ICC) of 0.9, and a significance of α = 0.05, the calculated sample size is 47. Taking into account dropouts, it was planned to include 50 participants [33].

#### 2.5.2. Data Analysis

Data analysis was conducted using SPSS, version 28. The significance level (α) was set at 5% (*p* < 0.05). Demographic and clinically relevant scar data at baseline were summarized using frequencies and the descriptive statistical measures of central location and dispersion. The heterogeneity of both groups was assessed using the Mann–Whitney U test (numeric variables, non-parametric), Pearson chi-square (categorical variables), and Independent Samples test (numeric variables, parametric). Following guidelines and prior research, equivalence or concordance was evaluated using intraclass correlation coefficients two-way mixed effects model for absolute agreement with 95% confidence intervals, ranging from 0 to 1, for all the POSAS individual items, overall opinion, and sum scores and for all the DLQI subscales and sum scores and EQ-5D mean VAS, EQ-5D index scores, and item scores. This is the most commonly used statistical analysis in equivalence studies of this nature [19]. For equivalence following Terwee et al. 2007, ICC > 0.7 is an acceptable agreement since we evaluate the comparator, the electronic versions, with the control, the paper versions [19,34]. In addition, the difference between paper and electronic items and sum scores within individual subjects were computed and median and interquartile range was calculated.

Bland–Altman analyses were also conducted to find out if the difference between MOAs falls between acceptable limits. The paper-based PROMs were plotted against e-PROMs. The score difference (paper minus electronic) was plotted against the average paper and electronic score for each individual, including 95% limits of agreement calculated by 1.96 × SD_difference_. Any systematic bias is thus separated from random measurement error.

Differences in completion times between the 2 MOAs were analyzed by comparing the means of the time measurements with the Paired Samples T Test. The Independent Samples test was used to compare the completion times of the paper and electronic versions between both groups to objectify a potential learning or memorization effect.

Data quality, which was defined as the number of incomplete responses and ambiguous data entries, was presented with a frequency table. The preference of the subjects towards an MOA was also summarized using frequencies. A potential difference between the 2 groups was analyzed with the Pearson chi-square test. The satisfaction questionnaire uses a 5-point Likert scale and was presented with a frequency distribution and a median as the measure of central tendency. The Kolmogorov–Smirnov test was used whenever necessary to test the normality assumption required by some statistical tests. The normality of the difference between variables or of the raw data was assessed for paired and non-paired analyses, respectively.

## 3. Results

### 3.1. Participants Flow

**Figure 3:**
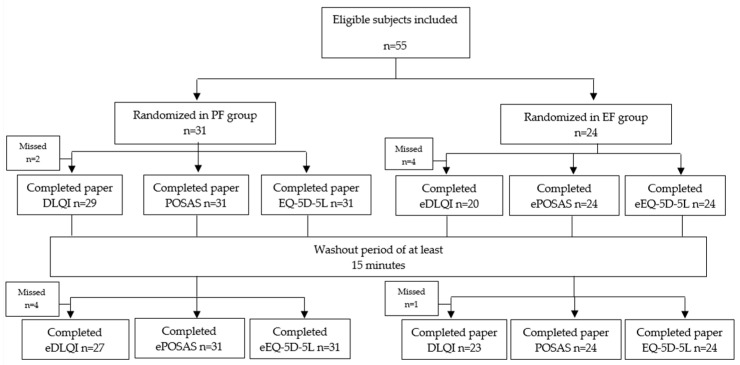
Participant flowchart. PF: paper-first EF: electronic-first.

A sample of 55 outpatients at our after-care center were enrolled. After randomization, the paper-first (PF) group consisted of 31 subjects, and the electronic-first (EF) group of 24 subjects. A flowchart demonstrating the participant flow is provided in Figure 3.

### 3.2. Baseline Data and Washout Period

The demographics and clinical characteristics of both groups are presented in Table 1. There were no statistical differences between the characteristics of both groups. The predetermined washout period between the different MOAs was at least fifteen minutes, the mean washout period was 24.8 min.

### 3.3. Equivalence of Scores

The ICC values ranged from 0.80 to 0.89 for all six POSAS items, the overall opinion, and the sum score of the POSAS. Thus, equivalence is assumed. The ICC values for DLQI ranged from 0.52 to 0.93. The treatment subscale of the DLQI (ICC = 0.52) had the lowest ICC. These results suggest that, by means of central tendency, the majority of the scores show equivalence between the electronic when comparing the electronic with the paper questionnaires and only the subscale Treatment of DLQI seemed not equivalent. For EQ-5D-5L, the ICC value was 0.95 for the EQ VAS score and 0.82 for the Index, showing equivalence of the electronic version of this questionnaire. For the individual items of the EQ-5D-5L, ICC ranged from 0.62 to 0.92, showing equivalence between the electronic and paper versions, and the item Pain/discomfort showed the lowest ICC, and the item Self-Care with 0.64 and the highest ICC values were found for EQ-5D VAS (0.95) and item Self-Care (0.92); the overall eEQ-5D seemed equivalent. Equivalence of scores is presented in Table 2.

### 3.4. Completion Times

The mean completion times can be found in Table 3. Interpreting the results, both PROMS were completed faster electronically but not significantly faster. The mean difference, however, not significant, is 4.33 s for DLQI and 2.13 s for POSAS in advantage of the electronic versions. For EQ-5D, the results of the paired samples T-test conclude that there is a significant difference between the two completion times (*p* < 0.01), the completion time of the electronic version is 12.96 s shorter than the completion time of the paper version.

A learning and memorization effect needed to be considered when interpreting completion times because, for example, a subject in the PF group was already exposed (e.g., remembers the questions) to the questionnaire when filling in the electronic version. One could, therefore, assume that the mean completion time of the second version would always be lower because of this earlier exposure. However, for all the questionnaires, the completion times of one MOA were not significantly different between groups (*p* > 0.05) as illustrated in Table 4. So, the order of completion did not matter with regard to completion time.

### 3.5. Data Quality

A number of incomplete responses and ambiguous data entries were registered. For the eDLQI and eEQ-5D-5L, incomplete responses were non-existent since the questionnaires had integrated controls implemented in the electronic variant that did not allow subjects to submit the questionnaire if questions were left open. For the paper version of the DLQI question, part 2 of question 7 was left open by three participants. For one paper version, the VAS score of the EQ-5D-5L was missing. The questions of the electronic version of the EQ-5D-5L were all complete because of a forced choice in the pathway. On paper six, the participants either wrote down the number or indicated the score on the scale. This means that the data were not technically missing, but the questionnaire was not filled in completely correctly. This automated control mechanism was not implemented in the ePOSAS which resulted in three incomplete responses.

Ambiguous data entry was present for all the paper questionnaires. For the POSAS, one subject ticked two answer options. The DLQI had four cases of ambiguous data entry. This was either, again, ticking two answer options (n = 2) or answering part 2 of question 7 when it was not necessary (n = 2). There was no impact on the scores for the DLQI. Ambiguous data entry was not possible in the electronic versions.

### 3.6. Preference and Satisfaction

For the question on the mode of preference, the preferred MOA was undoubtedly the electronic variant since 78.2% (n = 43) of the subjects preferred to fill in the electronic variants for future assessments. The other 16.4% (n = 9) had no preference and 5.5% (n = 3) preferred the paper mode. There was no difference in the preferred MOA between both groups (*p* = 1). More than 60% found that the education session at the beginning of the survey was helpful. A graphic representation of the scores to track the progression of their scar and quality of life would be an added value for more than 70% of the participants. More than 90% of the participants find electronic administration easy and a good idea. More than 70% of the participants found electronic administration easier or more practical.

The results of the satisfaction questionnaire, which asks the subjects about their thoughts and attitudes towards ePROMS with a 5-point Likert scale, were summarized in Table 5. For all but one item, there were no significant differences between both groups. The question “filling in paper questionnaires is faster than electronic” was, however, significantly different between both groups. An analysis of this difference showed a median score of three and two for the PF and EF group, respectively.

## 4. Discussion

This study employed a randomized crossover design aimed at assessing the equivalence between the traditional paper versions of the POSAS, EQ-5D-5L, DLQI, and their electronically formatted counterparts. The equivalence of the electronic questionnaires POSAS, EQ-5D-5L, and DLQI is assumed based on the findings of this study. Equivalence for all individual items, the overall opinion and sum scores of the POSAS, mean VAS and index of EQ-5D-5L, and all subscales and the total score of the DLQI was found. Only DLQI’s treatment subscale seems non-equivalent.

The feasibility of electronic versions is supported for their use in clinical practice and for research purposes in post-burn scar settings. Although completion time was observed to be a few seconds faster for the electronic versions of the POSAS and the DLQI, the difference did not reach statistical significance. The electronic EQ-5D-5L was completed significantly faster. Notably, in terms of data quality, the traditional paper format often exhibited instances of missing data attributed to various factors. Conversely, ePROMs offered the advantage of incorporating forced-choice options, thereby mitigating the occurrence of missing data. Regarding user preferences, the majority (78.2%) expressed a preference for the electronic versions. This preference underscores the potential for enhanced user satisfaction and engagement with electronic formats. The electronic version incorporated in electronic health records allows patients to complete questionnaires at home and prior to their consultations or treatments in outpatient settings. Both patients and clinicians could access this information during care pathways and allow the monitoring of changes in scar quality and quality of life over time in relation to the treatments received.

The present study supports the conclusions from other studies that generally speaking, PROMs obtained via paper or an electronic device yield comparable results, and thus are equivalent [18,20,35,36,37,38,39]. The recent decision flow chart regarding the need to establish measurement comparability [17] confirmed that in our case, the level of modification introduced by the migration was minor to moderate, and prior to this study, there was insufficient evidence demonstrating comparability between the modes of POSAS and EQ-5D-5L. Thus, in our situation, it was recommended that additional testing be considered to generate direct evidence of comparability between modes. The guidelines formulated by ISPOR state that in cases where sufficient evidence of measurement comparability exists and best practices for faithful migration are followed, no further testing of measurement comparability among the data collection modes is necessary. This includes cases of “mixing modes” within clinical trials, such as bring-your-own-device designs [17]. It is clinically reassuring that these results carry over to our population of (burn) scar patients.

All items, subscales, and sum scores indicated that the electronic delivery of the POSAS, EQ-5D, and DLQI are equivalent to their paper versions. The DLQI subscale ‘treatment’, scored only moderate, and this can be explained by the fact that the subscale only contains one question and all the other DLQI subscales combine two questions.

The EQ5-D-5L was completed significantly faster with a mean difference of 12.96 s in favor of the electronic version. It is reassuring that the completion times of all the electronic variants are at least equal or faster because filling in questionnaires requires cognitive effort and attention which may decrease as completion time increases.

One hypothesis to test was whether the second MOA was always completed faster since there had already been exposure to the questionnaire with the first MOA. A subject, therefore, could remember questions which would automatically result in completing the second MOA faster. This seemed to not be the case since, for all but one, the order of completion had no impact on the completion time. It did matter for the paper version of the DLQI, however (*p* < 0.05). Thus, the learning and memorization effect was limited partly because of the washout period. Our findings are supported by previous research regarding completion times being equal or faster in comparison with their paper-based counterparts [28,29,33,34].

Subject’s thoughts about the completion times were assessed in the satisfaction survey with a 5-point Likert scale. When stated “the time necessary to fill in electronic questionnaire was acceptable” 60% completely agreed, and the other 30% answered “neutral” or “agree”.

In theory, missing data or incomplete responses could be reduced to 0 with electronic assessments. This is because electronic assessment allows us to implement integrated controls where subjects are forced to fill in all the questions before they can submit their response. Thus, at its core, ePROMs are able to eliminate the problem of missing data [37,40,41] and enhance the integrity and accuracy of data [16]. As identified in a recent systematic review, data quality indeed tends to be higher when questionnaires are administered electronically [14]. In this study, integrated controls for checking that all necessary questions were filled in were implemented for the DLQI and EQ-5D-5L. This was purposely not performed for the POSAS. Consequently, the eDLQI and eEQ-5D-5L questionnaires had no incomplete responses and the ePOSAS had two.

Ambiguous data entries were present in at least one copy of all the paper questionnaires. This was either checking two answer boxes or answering a question that did not need to be answered (e.g., only answer if the previous question was “no”). The latter could be the result of a lack of cognitive attention and wanting to complete the questionnaire as fast as possible resulting in not reading the questions thoroughly. When ambiguous entries were encountered, the scoring guidelines were followed on how to handle these. In the electronic questionnaires, ambiguous entries were non-existent since it is not possible to check two answers, and questions that do not apply are automatically hidden for the subject.

Administration time is also reduced via ePROMs since automatic scoring can be embedded.

Our findings regarding the preferred MOA are complementary with other studies, being that the majority (78.2% in our study) of subjects prefer electronic administration for future assessments [36,37,38,40,42,43].

Thoughts and attitudes about electronic administration were assessed with a 5-point Likert scale with 1 being “completely disagree” and 5 “completely agree”. The ease of use of the electronic versions, measured with the statement “it was easy to fill in the electronic questionnaires”, was rated a median score of 5. Other items with a median score of 5 were “electronic administration is a good idea” and “the time necessary to fill in electronic questionnaires was acceptable”. Four items had a median score of 4, being “electronic administration and follow up of my health status/scar will improve quality of care”, “electronic administration was easier/more practical than paper administration”, “a graphical overview of my results to track my progress is an added value”, and “the educational session on how to operate the tablet was helpful”. This indicates that subjects were, on average, very supportive and satisfied regarding the electronic administration of questionnaires. The educational session on how to operate the tablet may not be necessary for everyone, but it is indispensable for certain subgroups of the population [38,44,45]. Prior experience with tablets, internet availability, education, and sociodemographic factors may influence preference [14].

It is important to bear in mind that the EQ-5D index score is not a patient-reported index and is designed for use in economic evaluations rather than clinical application [32]. In the DLQI, the patient-reported categorial items are converted to subscale scores and these subscale scores and DLQI sum score can no longer be seen as the patient’s direct response or as patient-reported outcome. For test–retest evaluation with both DLQI and POSAS measures on paper or electronic, within-responded item comparison can sometimes show differences whilst still having the same aggregated sum scores.

In the current study, the mean washout period was 24 min between subsequent MOA. This might be considered too short; however, other equivalence studies also used [42] a washout period of 30 min. The washout period was kept short to reduce patient time and travel burden and to avoid symptoms, severity, and complaints that did not fluctuate between administrations.

Note to mention that we used one device, one tablet, for the electronic delivery. In real-life settings, patients can bring their own device and or will fill out questionnaires via their smartphone or on a computer or tablet especially if performed remotely/from home. Bring your own device (BYOD), where patients use their own device for ePROM administration, is the ideal future step. There are, however, technical and practical considerations to take into account. BYOD may reduce costs and allow patients to work on familiar equipment. If sent by email or via an app, patients may turn off in-app notifications, remove the email or study app, change devices, run out of data or device storage, and be interrupted by other activities on the device [46]. Including only cognitively able and physically able patients with the majority of patients having internet and a tablet might have resulted in a sampling bias. This study was performed in an after-care setting, at Oscare in Belgium which might have caused bias. To the best of our knowledge, this is the first equivalence study comparing the electronic versions of commonly used PROMs in an adult population of patients with scars.

## 5. Conclusions

To conclude, following the recommendations of the ISPOR guidelines, this study is reassuring for the use of the electronic versions of the POSAS, EQ-5D-5L, and DQLI in the burns and scars setting for outpatient use and ePOSAS, eEQ-5D-5L, and eDLQI can be considered equivalent. Completion times for ePOSAS and eDLQI are equal or faster than their paper versions; especially for the eEQ-5D, significantly faster administration was reported. Scoring questionnaires electronically provides the benefit of scalability and having immediate results. Data quality and missing data can be reduced to 0 with electronic assessments. In addition, it was clear that electronic administration was feasible. Electronic versions were preferred, and thoughts and attitudes are very favorable towards ePROMs. This means a reassuring step towards a more widespread routine implementation of these measures and their positive effects on outpatient use. To start implementing electronic administration, we recommend providing an educational session and a testing period to detect issues that could potentially influence patient satisfaction, reliability, and validity. The use of these electronic measures can contribute to patient–professional dialog and better tailoring client-centered care.

## Figures and Tables

**Table 1:** Participant baseline demographics and clinical characteristics.

Description	PF Group (n = 31)	EF Group (n = 24)	Total (n = 55)
Gender, n (%)			
Male	22 (71%)	14 (58.3%)	36 (65.5%)
Female	9 (29%)	10 (41.7%)	19 (34.5%)
Age, mean ± SD (range)	39.00 ± 13.36 (19–70)	38.92 ± 17.60 (18–83)	38.96 ± 15.20 (18–83)
Cause of scar, n (%)			
Burn	15 (48.3%)	15 (62.5%)	30 (54.6%)
Non-Burn	16 (51.7%)	9 (37.5%)	25 (45.4%)
Access to internet, n (%)			
Yes	31 (100%)	23 (95.8%)	54 (98.2%)
No	0 (0.0%)	1 (4.2%)	1 (1.8%)
Level of education, n (%)			
No education	1 (3.2%)	3 (12.5%)	4 (7.3%)
Secondary school	12 (38.7%)	13 (54.2%)	25 (45.5%)
Bachelor	13 (41.9%)	6 (25.0%)	19 (34.5%)
Master	4 (12.9%)	2 (8.3%)	6 (10.9%)
Doctor	1 (3.2%)	0 (0.0%)	1 (1.8%)
Tablet at home, n (%)			
Yes	23 (74.2%)	20 (83.3%)	43 (78.2%)
No	8 (25.8%)	4 (16.7%)	12 (21.8%)

**Table 2:** ICC values for the agreement between the paper and electronic versions of POSAS DLQI and EQ-5D-5L.

PROMS Item or Subscale (n of Questions)	Paper fg	Electronic	ICC (*p*)	Difference P-E
	Mean (SD)	Mean (SD)		Median (IQR)
POSAS				
Pain (1)	3.09 (2.34)	3.42 (2.42)	0.85 (*p* ≤ 0.001)	−0.32 (−1–0)
Itch (1)	3.69 (2.60)	3.70 (2.83)	0.80 (*p* ≤ 0.001)	0 (0–0)
Color (1)	5.98 (2.33)	6.07 (2.52)	0.80 (*p* ≤ 0.001)	0 (0–1)
Stiffness (1)	5.73 (2.92)	5.65 (2.73)	0.80 (*p* ≤ 0.001)	0 (0–1)
Thickness (1)	5.59 (2.74)	5.43 (2.74)	0.84 (*p* ≤ 0.001)	0 (0–1)
Irregularity (1)	5.31 (2.76)	5.17 (2.74)	0.86 (*p* ≤ 0.001)	0 (0–1)
POSAS Overall opinion (1)	5.27 (2.28)	5.1 (1.8)	0.89 (*p* = 0.000)	0 (−2–1.75)
POSAS sum score (6)	29.92 (12.85)	30.5 (11.1)	0.84 (*p* ≤ 0.001)	0 (0–1)
DLQI	Median (IQR)	Median (IQR)		
Symptoms and feelings (2)	1 (0–3)	1 (0–3)	0.76 (*p* ≤ 0.001)	0 (−1–0)
Daily activities (2)	1 (0–3)	1 (0–3)	0.77 (*p* ≤ 0.001)	0 (0–1)
Leisure (2)	1 (0–3)	1 (0–3)	0.82 (*p* ≤ 0.001)	0 (0–0)
Work and school (1)	1 (0–3)	0 (0–1)	0.94 (*p* ≤ 0.001)	0 (0–0)
Personal relationships (2)	0 (0–1)	0 (0–1)	0.84 (*p* ≤ 0.001)	0 (0–0)
Treatment (1)	0 (0–1)	0 (0–1)	0.52 (*p* ≤ 0.001)	0 (0–0)
DLQI sum score (10)	6.5 (1–12)	5 (1–12)	0.93 (*p* ≤ 0.001)	0.04 (−1–2)
EQ-5D-5L	Mean (SD)	Mean (SD)		
EQ VAS	74.28 (17.34)	75.20 (17.08)	0.95 (*p* < 0.001)	0 (0–1)
Index	0.86 (0.16)	0.84 (0.17)	0.82 (*p* < 0.001)	0 (0–0.03)
	Median (IQR)	Median (IQR)		
Mobility (1)	1 (1–1)	1 (1–1)	0.79 (*p* < 0.001)	0 (0–0)
Self-care (1)	1 (1–1)	1 (1–1)	0.92 (*p* = 0.000)	0 (0–0)
Usual activities (1)	1 (1–3)	1 (1–3)	0.85 (*p* = 0.000)	0 (0–0)
Pain Discomfort (1)	1 (1–2)	1 (1–3)	0.62 (*p* < 0.001)	0 (0–0)
Anxiety depression (1)	1 (1–2)	1 (1–2)	0.75 (*p* < 0.001)	0 (0–0)

Scatter plots for all 6 POSAS items and EQ-5D VAS together with contingency tables for DLQI and EQ-5D-5L were added in Supplementary File S1. The Bland–Altman plots for the POSAS overall opinion and sum score, the EQ-5D-5L VAS and index, and the DLQI sum score for the comparison of the paper version and electronic are presented in the Supplementary File S1.

**Table 3:** Overview result analysis of completion times.

Questionnaire	N	Mode of Administration	Mean	Mean Difference (s)	Significance
DLQI	54	Electronic	93.24 s	4.33	0.408
		Paper	97.57 s		
POSAS	55	Electronic	58.28 s	2.13	0.415
		Paper	60.41 s		
EQ-5D	55	Electronic	54.27 s	12.96	0.002
		Paper	67.24 s		

**Table 4:** Overview result analysis of possible learning curve.

Questionnaire	Group	Electronic Version			Paper Version		
		Mean (s)	Mean Difference (s)	Significance	Mean	Mean Difference	Significance
DLQI	Paper first	86.93	14.20	0.281	108.00	22.83	0.05
	Electronic first	101.13			85.17		
POSAS	Paper first	57.21	2.47	0.78	64.38	9.087	0.27
	Electronic first	59.68			55.29		
EQ-5D-5L	Paper first	49.32	11.34	0.21	78.23	25.18	0.02
	Electronic first	60.67			53.04		

**Table 5:** Results for the satisfaction questionnaire.

Questions	Likert Scale ^a,b^					
	1	2	3	4	5	Median
It was easy to fill in the electronic questionnaire	1 (1.8)	0 (0)	4 (7.3)	17 (30.9)	33 (60)	5
Electronic administration of questionnaires is a good idea	2 (3.6)	0 (0)	3 (5.5)	17 (30.9)	33 (60)	5
Electronic administration was easier/more practical than paper administration	1 (1.8)	4 (7.3)	11 (20)	18 (32.7)	21 (38.2)	4
Electronic administration and follow-up of my health status/scar will improve my quality of care	1 (1.8)	1 (1.8)	12 (21.8)	23 (41.8)	18 (32.7)	4
The time necessary to fill in electronic questionnaires is acceptable	1 (1.8)	0 (0)	3 (5.5)	18 (32.7)	33 (60)	5
Filling in paper questionnaires is faster than electronic	8 (14.5)	19 (34.5)	19 (34.5)	5 (9.1)	4 (7.3)	3
The educational session on how to operate the tablet and fill in the electronic versions was helpful	3 (5.5)	4 (7.3)	12 (21.8)	26 (47.3)	10 (18.2)	4
A graphical overview of my results is an added value to track the progress of my scar and health	1 (1.8)	1 (1.8)	13 (23.6)	16 (29.1)	24 (43.6)	4

^a^ 1 = completely disagree; 2 = disagree; 3 = neutral; 4 = agree; 5 = completely agree; ^b^ n (%).

## Data Availability

The participants of this study did not give written informed consent for their data to be shared publicly, so due to that and the sensitive nature of the information, data are not available.

## Supplementary Materials

The following supporting information can be downloaded at: https://www.mdpi.com/article/10.3390/ebj5040030/s1, Figure S1: Scatter plots of ratings for the 6 POSAS items, Table S1: Contingency tables showing the differences (within patients) between modes for DQLI subscales, Table S2: ICC values for the agreement between paper and electronic versions of DLQI items, Figure S2: Scatter plots of ratings for the EQ-5D VAS.
