# Supplementary material for: Measurement Equivalence and Feasibility of the Electronic and Paper Versions of the POSAS, EQ-5D, and DLQI: A Randomized Crossover Trial
Source: Eur Burn J. 2024 Oct 11;5(4):321–34. doi: 10.3390/ebj5040030 (PMC11727002; doi:10.3390/ebj5040030)
Supplement: Supplementary file 1 [file ebj-05-00030-s001.zip › ebj-3154060-supplementary.pdf]

Supplementary file S1

Figure S1 Scatter plots of ratings for the 6 POSAS items

A. POSAS pain

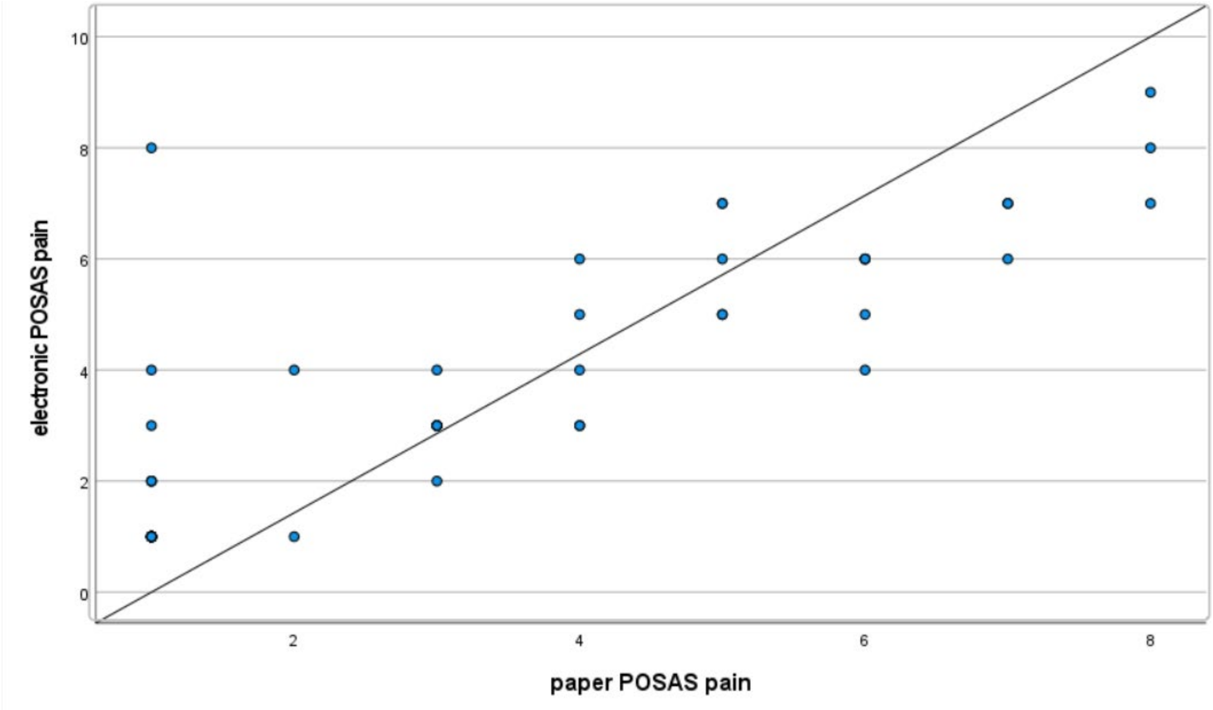

B. POSAS itch

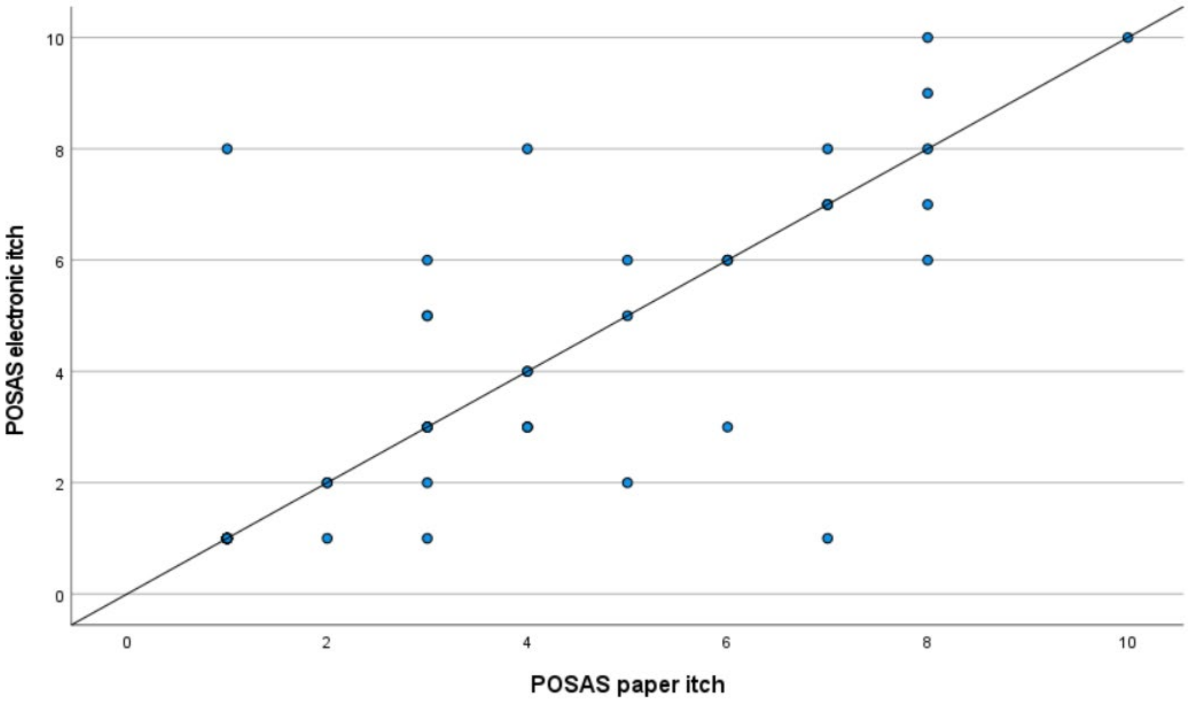

C. POSAS color

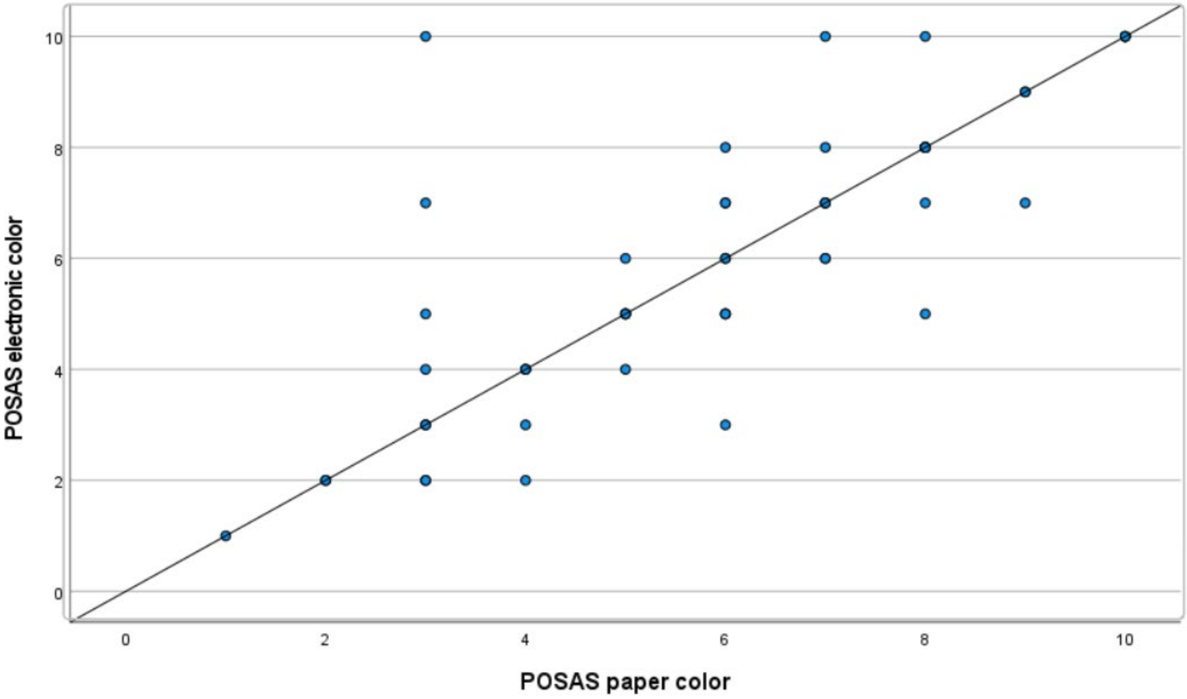

D. POSAS Stiffness

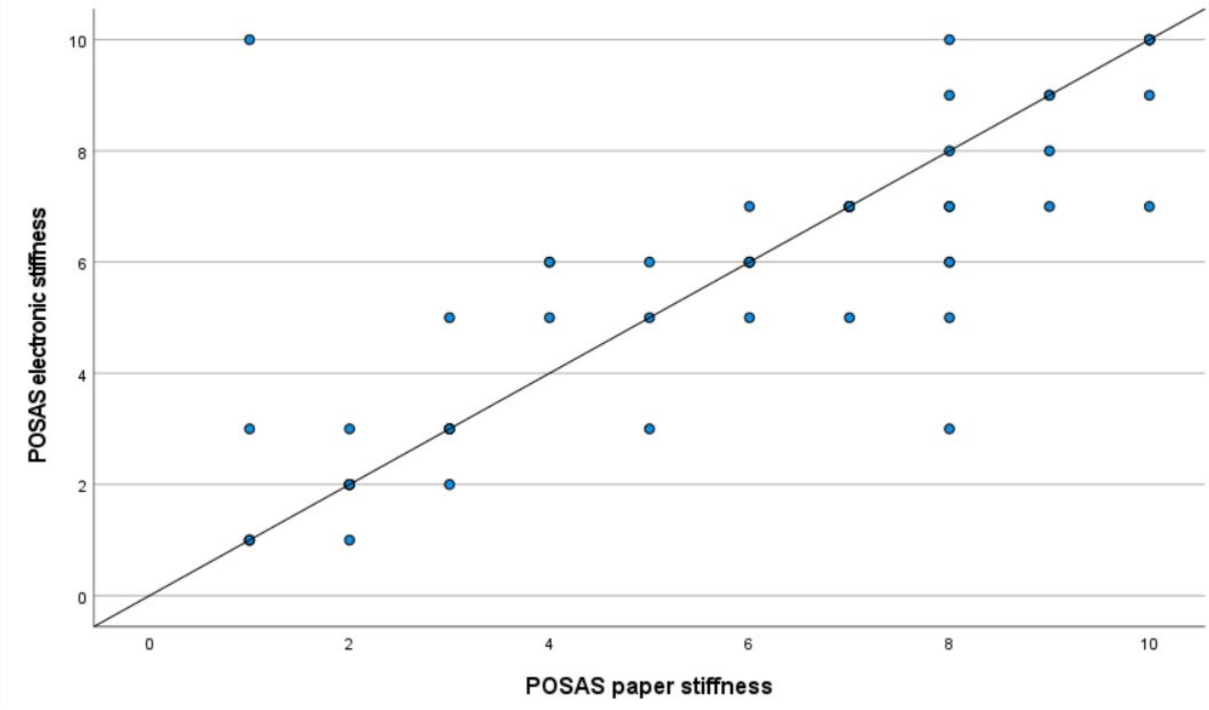

E. POSAS Thickness

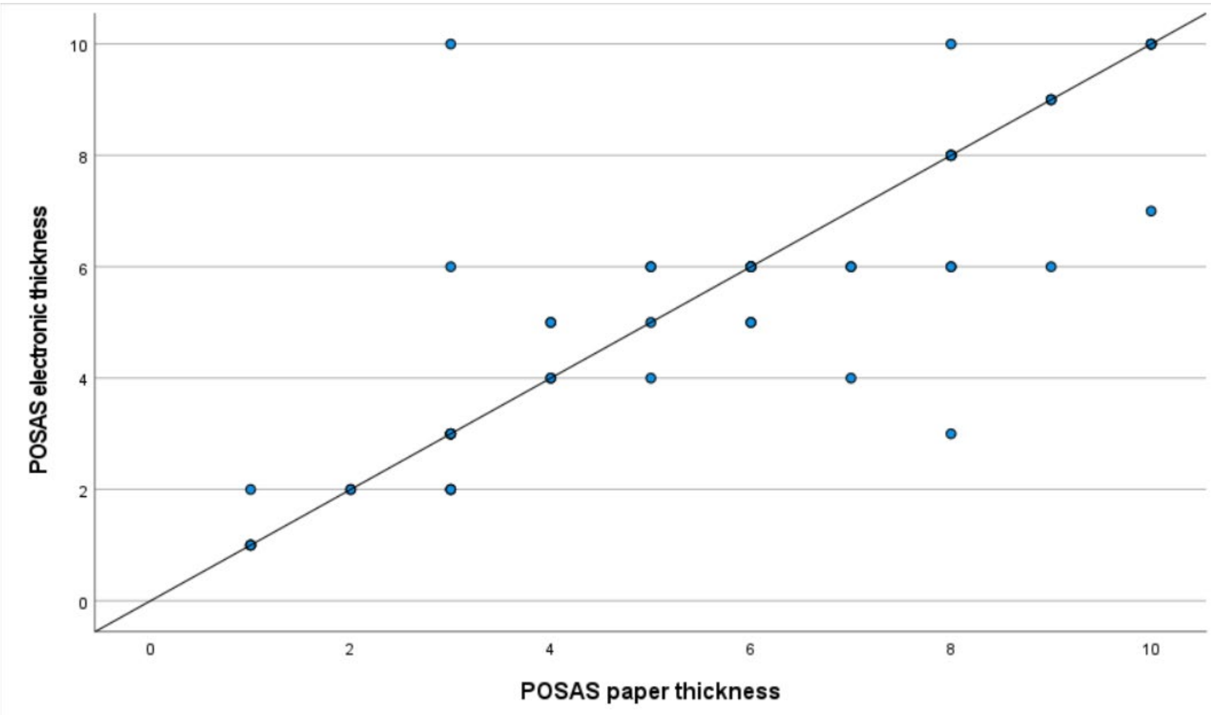

F. POSAS Irregularity

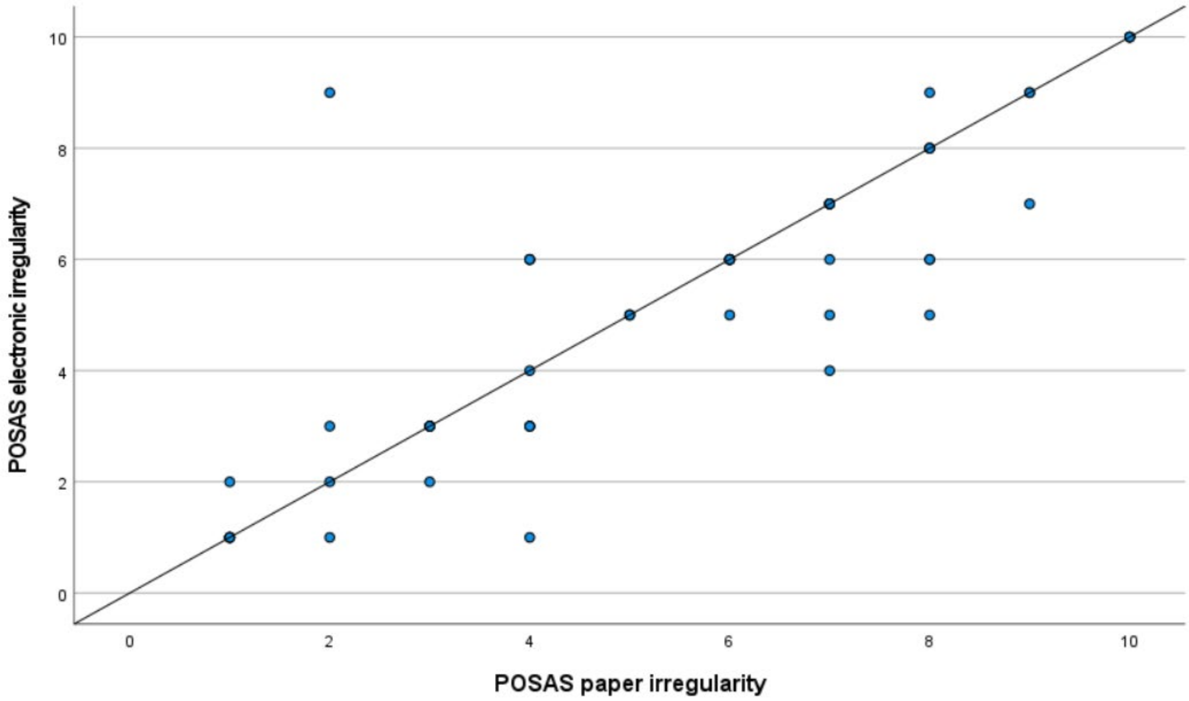

Table S1. Contingency tables showing the differences (within patients) between modes for DQLI subscales

		Electronic subscale Symptoms and feelings						
		0	1	2	3	4	5	Total
Paper subscale Symptoms and feelings	0	13	0	1	0	1	0	15
	1	3	9	2	0	0	0	14
	2	0	2	5	4	1	0	12
	3	1	2	0	2	2	0	7
	4	0	0	0	1	3	3	7
	Total	17	13	8	7	7	3	55

		Electronic subscale Daily activities							
		0	1	2	3	4	5	6	Total
Paper subscale Daily Activities	0	18	0	0	1	0	0	0	19
	1	6	7	2	0	0	0	0	15
	2	0	1	1	3	0	0	0	5
	3	0	1	3	3	1	2	0	10
	4	0	0	1	1	0	0	0	2
	5	1	0	0	0	1	1	0	3
	6	0	0	0	0	0	0	1	1
	Total	25	9	7	8	2	3	1	55

		Electronic subscale Leisure							
		0	1	2	3	4	5	6	Total
Paper subscale Leisure	0	21	0	0	2	0	0	0	23
	1	5	3	2	0	0	0	0	10
	2	0	0	4	2	0	0	0	6
	3	0	0	1	5	1	0	0	7
	4	0	0	0	1	1	0	1	3
	5	0	0	0	1	0	2	0	3
	6	0	0	1	1	0	1	0	3
	Total	26	3	8	12	2	3	1	55

		Electronic subscale Work and school			
		0	1	3	Total
Paper subscale Symptoms and feelings	0	22	3	0	25
	1	1	3	1	5
	3	0	1	16	17
	Total	23	7	17	47

		Electronic subscale Personal relationships					
		0	1	2	3	5	Total
Paper subscale Personal relationships	0	34	3	0	1	0	38
	1	2	4	0	0	0	6
	2	0	2	4	1	0	7
	3	1	0	1	0	1	2
	4	0	0	0	1	1	2
	Total	36	9	5	3	2	55

		Electronic subscale Treatment				
		0	1	2	3	Total
Paper Treatment	0	27	1	1	1	30
	1	3	11	5	1	20

	2	0	0	4	0	4
	3	1	0	0	0	4
	Total	31	12	10	2	1

Table S2 ICC values for the agreement between paper and electronic versions of DLQI items

**DLQI**	Median (IQR)	Median (IQR)	
Question 1	1 (0-1)	1 (0-1)	0.64 (p<.001)
Question 2	1 (0-1)	1 (0-1)	0.73 (p<.001)
Question 3	1 (0-1)	0 (0-1)	0.71 (p<.001)
Question 4	0 (0-1)	0 (0-1)	0. 62 (p<.001)
Question 5	0 (0-1)	0 (0-1)	0.57 (p<.001)
Question 6	0 (0-2)	0 (0-2)	0.94 (p=.000)
Question 7	0 (0-0)	0 (0-0)	0.43 (p=.001)
Question 8	0 (0-1)	0 (0-1)	0.60 (p<.001)
Question 9	0 (0-0)	0 (0-1)	0.89 (p=.000)
Question 10	0 (0-1)	1 (0-1)	0.64 (p<.001)

Figure S2 Scatter plots of ratings for the EQ-5D VAS

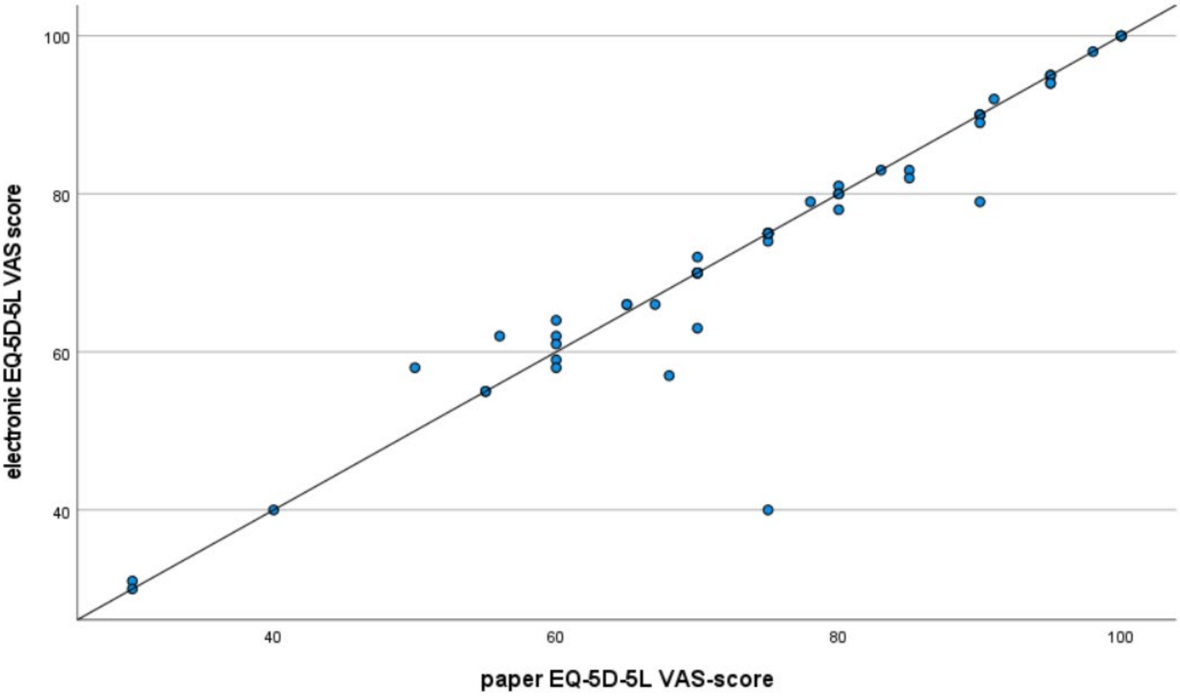

Supplementary material Bland Altmann plots

Bland Altmann plot POSAS sum score

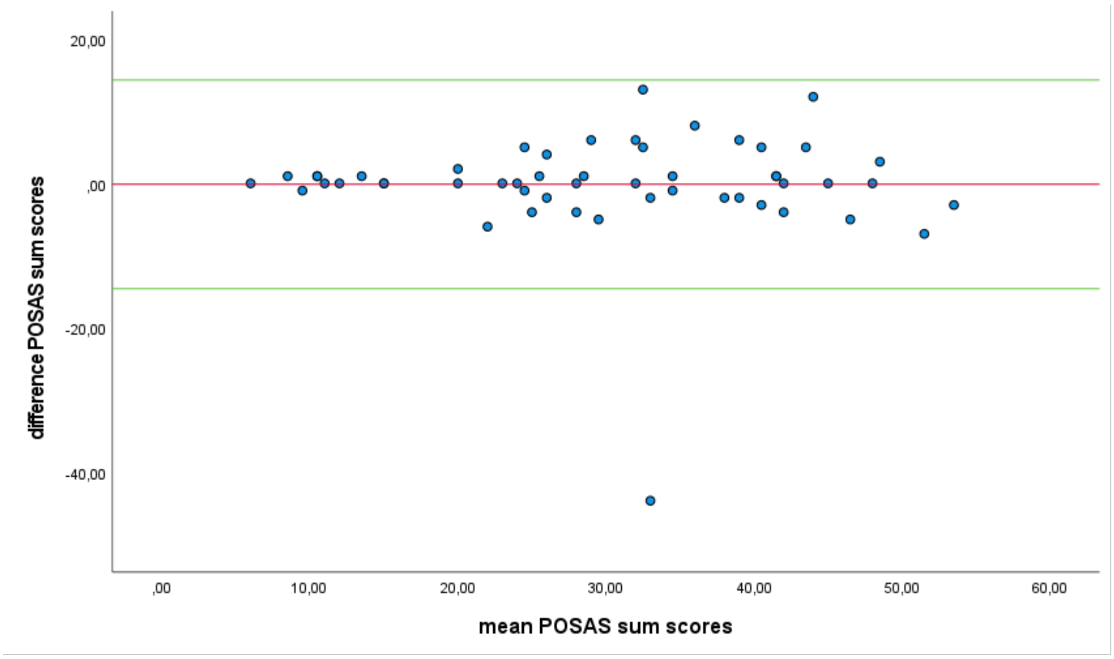

Bland Altmann plot POSAS overall opinion

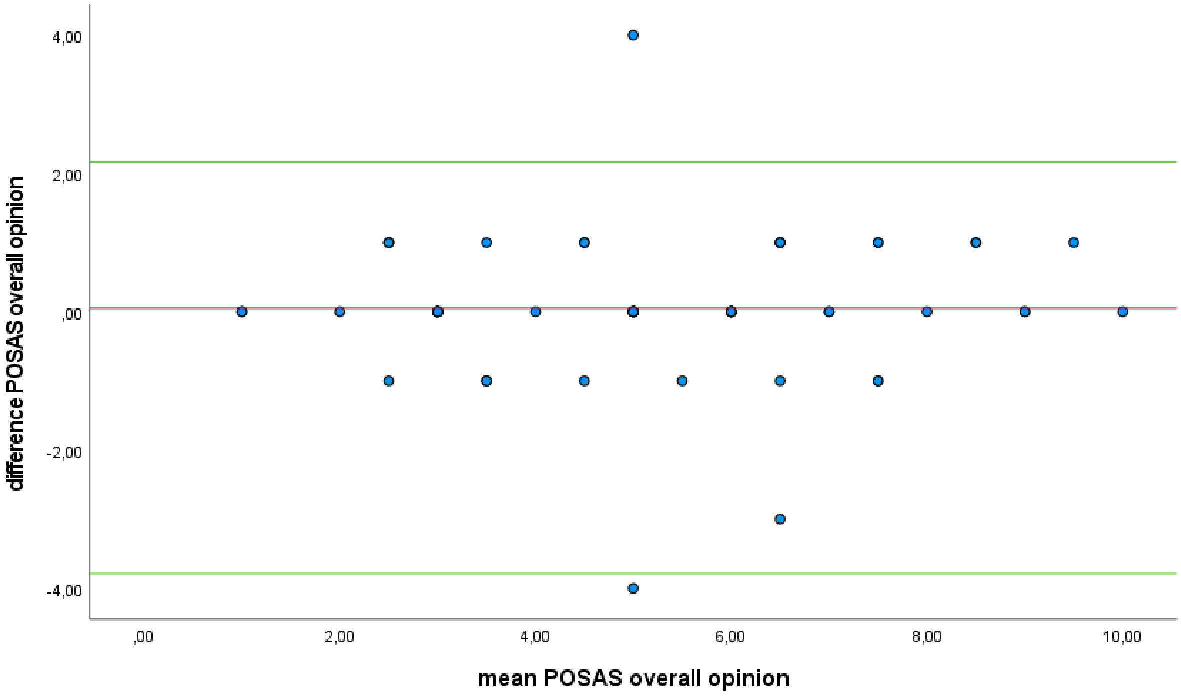

Bland Altmann plot EQ-5D-5L VAS score

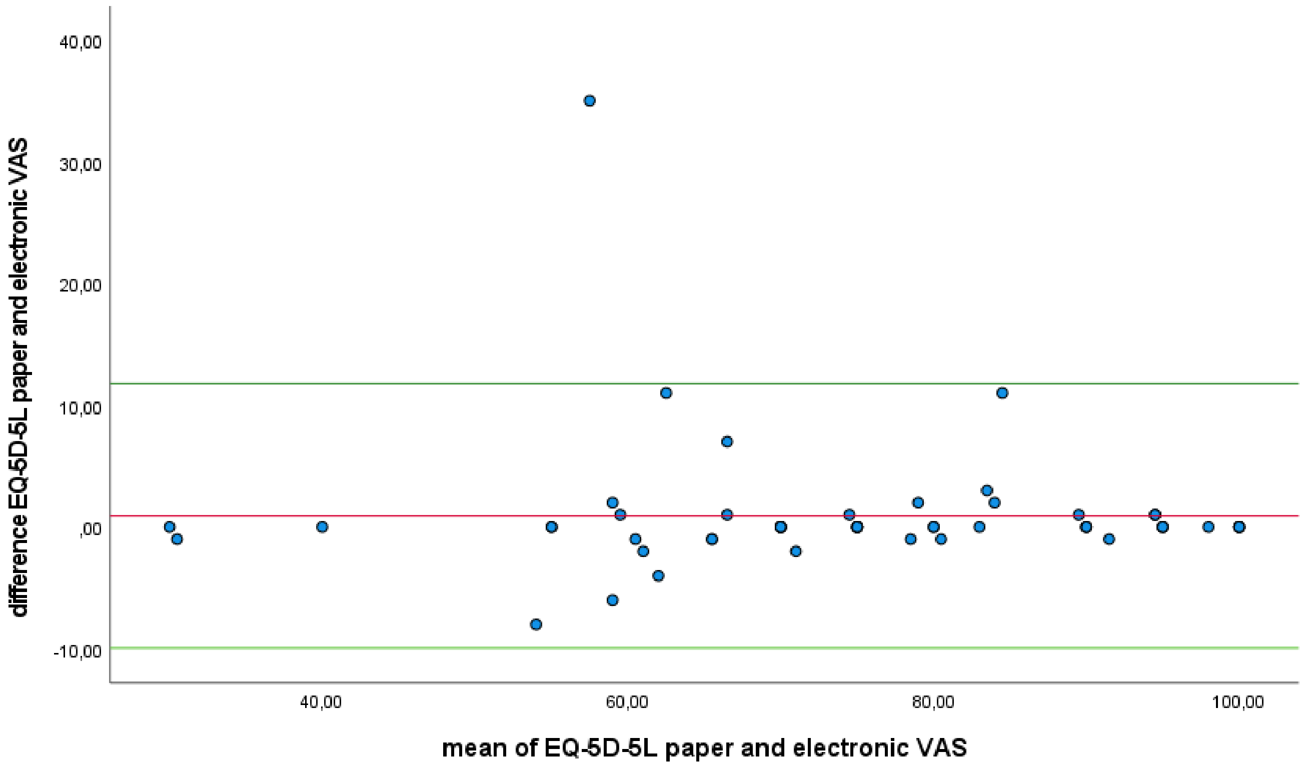

Bland Altmann plot EQ-5L index score

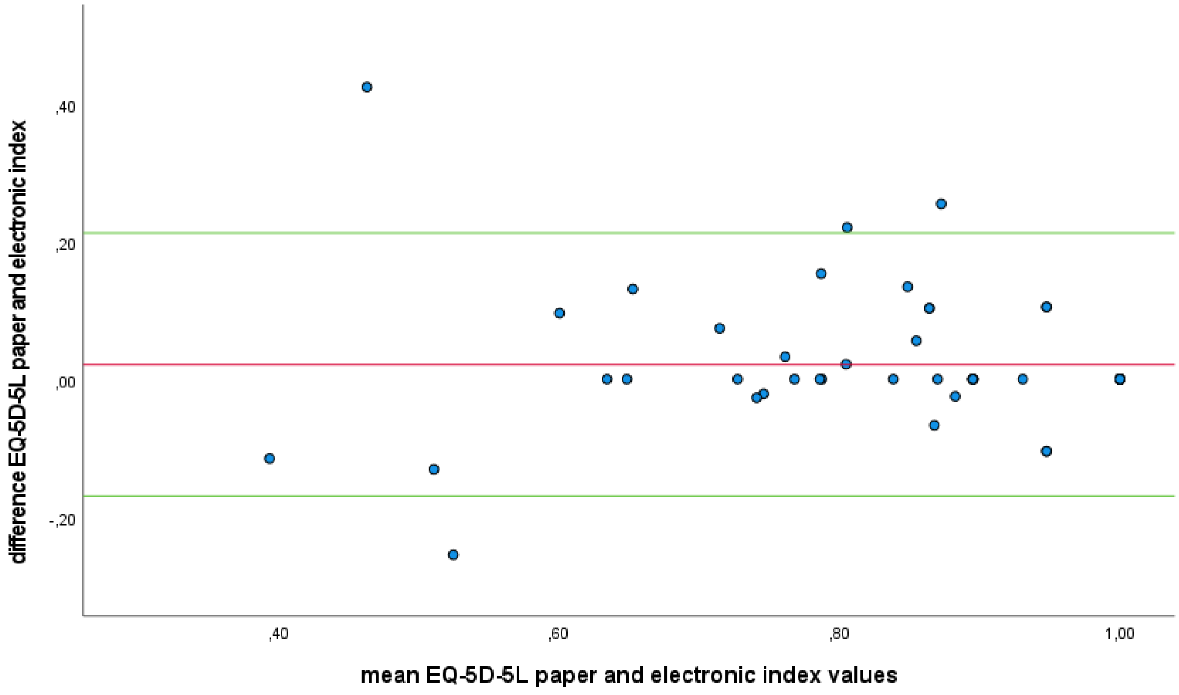

Bland Altmann plots DLQI sum score

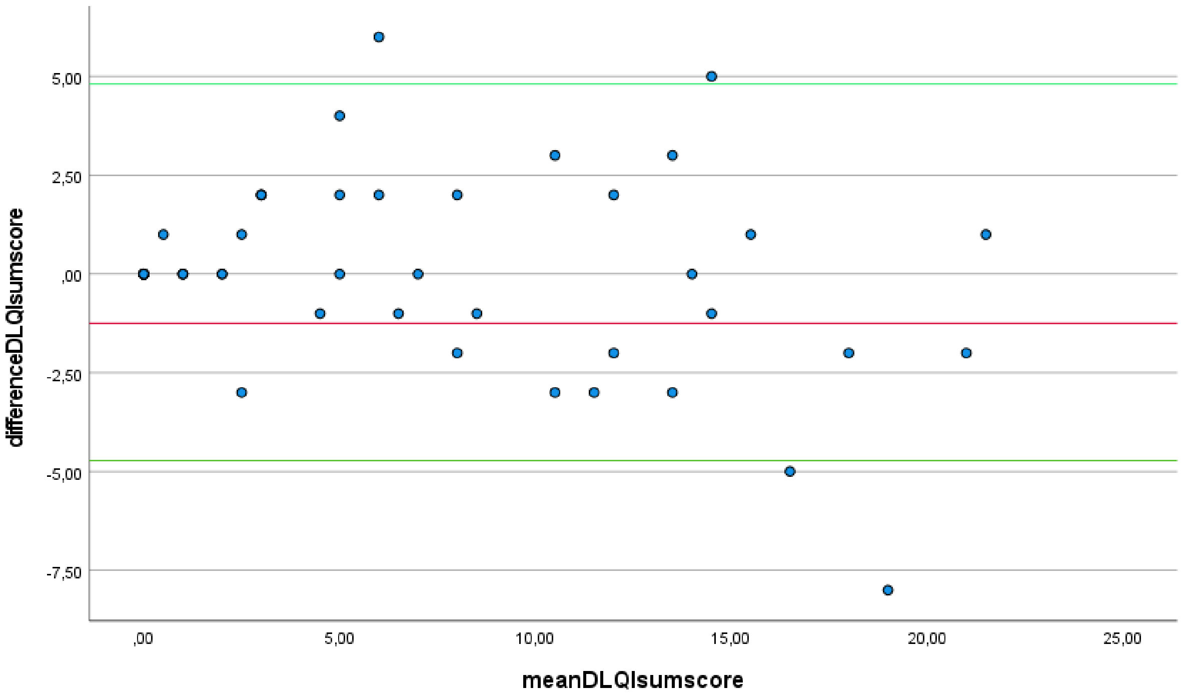
5,00
2,50
,00
-2,50
-5,00
-7,50
differenceDLQIsumscore
,00
5,00
10,00
15,00
20,00
25,00
meanDLQIsumscore
